# Epac, Rap and Rab3 act in concert to mobilize calcium from sperm’s acrosome during exocytosis

**DOI:** 10.1186/s12964-014-0043-0

**Published:** 2014-08-27

**Authors:** María C Ruete, Ornella Lucchesi, Matías A Bustos, Claudia N Tomes

**Affiliations:** Instituto de Histología y Embriología IHEM-CONICET, Facultad de Ciencias Médicas, Universidad Nacional de Cuyo, 5500 Mendoza, Argentina

**Keywords:** Acrosome, Calcium, cAMP, Epac, Exocytosis, PTP1B, Rab3, Rap, Secretory granule, Sperm

## Abstract

**Background:**

Exocytosis of sperm’s single secretory granule or acrosome (acrosome reaction, AR) is a highly regulated event essential for fertilization. The AR begins with an influx of calcium from the extracellular milieu and continues with the synthesis of cAMP and the activation of its target Epac. The cascade bifurcates into a Rab3-GTP-driven limb that assembles the fusion machinery and a Rap-GTP-driven limb that mobilizes internal calcium.

**Results:**

To understand the crosstalk between the two signaling cascades, we applied known AR inhibitors in three experimental approaches: reversible, stage-specific blockers in a functional assay, a far-immunofluorescence protocol to detect active Rab3 and Rap, and single cell-confocal microscopy to visualize fluctuations in internal calcium stores. Our model system was human sperm with their plasma membrane permeabilized with streptolysin O and stimulated with external calcium. The inhibition caused by reagents that prevented the activation of Rap was reversed by mobilizing intracellular calcium pharmacologically, whereas that caused by AR inhibitors that impeded Rab3’s binding to GTP was not. Both limbs of the exocytotic cascade joined at or near the stage catalyzed by Rab3 in a unidirectional, hierarchical connection in which the intra-acrosomal calcium mobilization arm was subordinated to the fusion protein arm; somewhere after Rab3, the pathways became independent.

**Conclusions:**

We delineated the sequence of events that connect an external calcium signal to internal calcium mobilization during exocytosis. We have taken advantage of the versatility of the sperm model to investigate how cAMP, calcium, and the proteinaceous fusion machinery coordinate to accomplish secretion. Because the requirement of calcium from two different sources is not unique to sperm and fusion proteins are highly conserved, our findings might contribute to elucidate mechanisms that operate in regulated exocytosis in other secretory cell types.

**Electronic supplementary material:**

The online version of this article (doi:10.1186/s12964-014-0043-0) contains supplementary material, which is available to authorized users.

## Background

Mammalian fertilization depends on special types of calcium-regulated exocytosis undergone by both the male and female gametes. Exocytosis of sperm’s single, very large dense core granule or acrosome is termed the acrosome reaction (AR). The AR is governed by a proteinaceous fusion machinery highly conserved among all organelles and organisms [1,2]. This machinery includes members of the secretory Rab and SNARE superfamilies as well as their interacting proteins and regulators. Prior to bilayer mixing, both Rabs and SNAREs assemble in multiprotein complexes that constitute molecular bridges between the membranes that are going to fuse [3-5]. The AR also relies on an increase in the cytosolic concentration of calcium via both an influx from the extracellular milieu through plasma membrane channels and an efflux from an internal store through inositol 1, 4, 5 triphosphate (IP_3_)-sensitive channels [6,7]. The former can be bypassed when exocytosis is triggered by inducers whose targets are situated downstream the opening of plasma membrane calcium channels in the signaling cascade, for instance cAMP [8], phorbol 12-myristate 13-acetate [9], and recombinant, active Rab3A [10]; such agonists elicit exocytosis even in the absence of extracellular calcium. Intracellular calcium mobilization cannot be bypassed without compromising exocytosis [11].

Cyclic AMP is both a pharmacological AR inducer and a second messenger produced endogenously by sperm. A soluble adenylyl cyclase (sAC) synthesizes the cAMP required for the AR [12]. The target of cAMP relevant to sperm exocytosis is the exchange protein directly activated by cAMP (Epac) [8]. Epacs are multi-domain proteins whose C-terminal catalytic region bear a guanine-nucleotide exchange factor (GEF) activity specific for Rap1 and Rap2 [13,14]. The exchange of GDP for GTP on Rap1 is mandatory to accomplish the AR [12]. The end point of the Rap-driven signaling pathway is the mobilization of intracellular calcium through IP_3_-sensitive channels.

**Figure 1:**
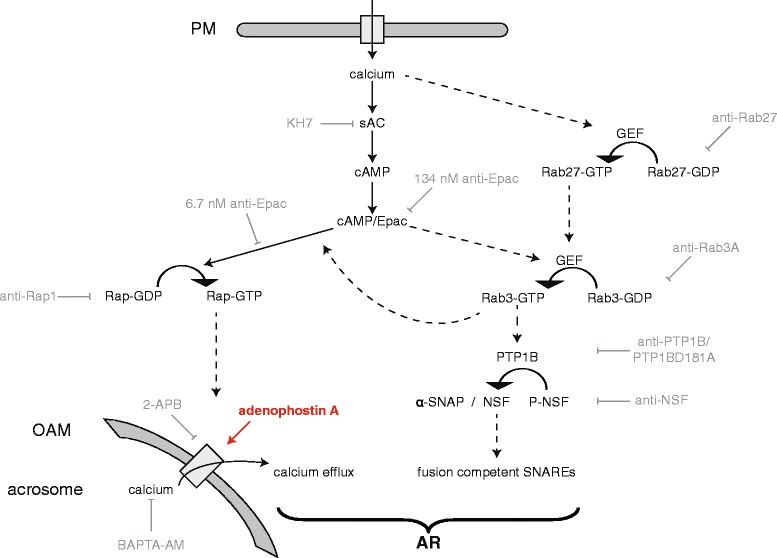
**Updated working model for the AR showing a bifurcated signaling pathway downstream of cAMP/Epac.** Calcium enters the cell from the extracellular milieu through the SLO-generated pores and activates sAC. Calcium also stimulates, directly or indirectly, the exchange of GDP for GTP on Rab27. Cyclic AMP synthesized by sAC activates Epac and here the signaling pathway splits into two limbs. In one of them, Epac catalyzes the exchange of GDP for GTP on Rap; in the other, cAMP/Epac indirectly activates Rab3. Rap-GTP heads a pathway that leads to acrosomal calcium mobilization (“calcium efflux” in the Figure). Rab3-GTP heads a pathway that leads to the correct assembly of the fusion machinery (“fusion competent SNAREs” in the Figure). Somewhere downstream of Rab3-GTP, there is a unidirectional (Rab3 limb → Rap1 limb) connection between both arms of the pathway. After this point, PTP1B is activated and/or recruited to the sites where it dephosphorylates NSF, derepressing its activity. Next, active, dephospho-NSF, in a complex with α-SNAP, renders SNARE proteins fusion competent. The step catalyzed by active SNAREs converges with the local increase in calcium coming from the acrosome downstream of Rap-GTP to accomplish the final steps of membrane fusion (“AR” in the Figure). Reversible and irreversible AR blockers used in this manuscript are shown in light gray. Adenophostin A (red) binds IP_3_-sensitive channels and mobilizes acrosomal calcium pharmacologically. PM, plasma membrane; OAM, outer acrosomal membrane. Solid arrows mean there is one step between the terms connected, dashed arrows mean that the number of steps is either unknown or not depicted for simplicity. Modified from [12].

A few years ago we put forth a model for the AR signaling cascade in which Epac occupies a central point (Figure 1). We had suggested that upstream the activation of Epac by cAMP there is a linear sequence of reactions; downstream of cAMP/Epac the pathway bifurcates into two limbs. One limb, termed the fusion machinery limb, contains Rab3A, PTP1B, α-SNAP/NSF, and SNAREs. Its function is to assemble the fusion machinery into place so that the outer acrosomal and plasma membranes become physically connected. The other, termed the calcium mobilization limb, contains Rap1 and a phospholipase C. Its purpose is to mobilize intracellular calcium through IP_3_-gated channels [12]. IP_3_ is generated when phospholipase Cs hydrolyze phosphatidylinositol-4,5-biphospate. The other product of this reaction is diacylglycerol; this lipid engages in a positive feedback loop that contributes to maintain the production of IP_3_ [9]. Here, we report fundamental advances in our understanding on how do these pathways coordinate to achieve the AR. We employed an exocytosis assay conducted with stage-specific reversible blockers and a novel far-immunofluorescence assay to detect how perturbing different members of the signaling cascade affects the activation status of Rab3 and Rap. We also visualized the emptying of the acrosomal calcium store in response to external calcium by single cell confocal microscopy and determined how AR blockers influence this release. The data reported here show that both limbs of the exocytotic cascade are joined at or near the stage catalyzed by Rab3 in a unidirectional, hierarchical connection in which the intra-acrosomal calcium mobilization arm is subordinated to the fusion protein arm; somewhere after Rab3, the pathways become independent.

## Results and discussion

### A novel protocol detects active Rap in the acrosomal region of human sperm

The signaling pathways underlining the stages of the AR that follow the influx of calcium from the extracellular milieu are quite complex. They begin with the production of cAMP and the activation of its target Epac. Afterwards, the cascade splits into two limbs, one that mobilizes intracellular calcium and another that activates the fusion machinery so that the acrosome becomes docked to the plasma membrane [12,15]. We have known for a few years that the activation of the small GTPase Rap is instrumental to accomplish the calcium mobilization limb [9,12]. We became interested in determining the percentage of the sperm population that exhibits activated Rap in response to an AR inducer, and in identifying where in the cell this active Rap is localized. Pull-down assays are not suitable for addressing these issues; thus, we applied a protocol that combines the protein-protein interaction principles of Far-western blot technology with immunodetection by fluorescence microscopy [16]. We had described the method for Rabs 27 and 3 and here we extended it to Rap. To determine the method’s suitability to determine Rap’s activation status, we incubated streptolysin-O (SLO)-permeabilized sperm with EDTA to chelate endogenous Mg^2+^ cations and allow for the release of endogenous guanosine nucleotides. Next, we loaded the cells with GDP-β-S or GTP-γ-S. Last, we fixed and smeared sperm on slides, overlayed with the protein cassette GST-Ral-GDS-RBD that binds Rap-GTP, washed and proceeded as for standard immunostaining using anti-GST antibodies to detect the activity probe. The population of cells immunodecorated with the anti-GST antibodies in the acrosomal region was higher when sperm were loaded with GTP-γ-S than when they were loaded with GDP-β-S (Additional file 1).

Thus, we have validated our protocol by demonstrating that Ral-GDS-RBD can distinguish between GDP- and GTP-bound Rap. Furthermore, GTP-bound Rap localized to the acrosomal region — the pattern expected for proteins with a role in exocytosis — in a large subpopulation of cells.

### Influence of agents that perturb the AR on the activation status of Rap and Rab3

Epac plays a central role in the activation of small GTPases during the AR. So much so that the Epac selective cAMP analogue 8-pCPT-2'-O-Me-cAMP increases the amount of active Rab3 and Rap pulled down from human sperm compared to untreated controls [12,16]. Because of the central position occupied by Epac in the exocytotic cascade (Figure 1), we considered essential to gather additional data to validate its role in driving the two downstream pathways described before.

**Figure 2:**
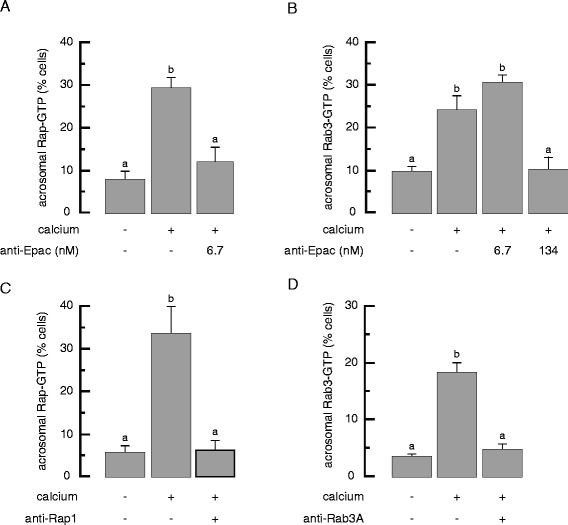
**The activation of endogenous Rab3 and Rap triggered by external calcium is differentially sensitive to Epac-sequestering antibodies.** SLO-permeabilized sperm were treated with 100 μM 2-APB and, when indicated, 6,7 **(A, B)**, or 134 nM **(B)** anti-Epac, 134 nM anti-Rap1 **(C)**, or 70 nM anti-Rab3A **(D)** antibodies. The AR was initiated with 0.5 mM CaCl_2_. Incubations were for 15 min at 37°C after each addition. Samples were processed for far-immunofluorescence as described in the Methods section, using GST-Ral-GDS-RBD **(A, C)** or GST-RIM-RBD **(B, D)** as activity probes. Shown is the percentage of cells immunodecorated in the acrosomal region with the anti-GST antibodies, representing active Rap **(A, C)** and Rab3 **(B, D)**. Plotted is the mean ± SEM of at least three independent experiments. Different letters indicate statistical significance (A and B, P < 0.01; C, P < 0.05; D, P < 0.001).

Based on the ability of adenophostin A — an IP_3_ receptor agonist expected to promote internal calcium release — to rescue the AR, we had hypothesized that 134 nM anti-Epac antibodies would impair the activation of both Rap and Rab3, whereas 6.7 nM anti-Epac would prevent the exchange of GDP for GTP on Rap but not on Rab3 [12]. In other words, low anti-Epac antibody concentrations would impair only the calcium mobilization limb whereas higher concentrations would interfere with calcium mobilization as well as with the fusion machinery limb. We conducted far-immunofluorescence experiments to test these premisses directly and obtained results that fully supported our model (Figure 1). Our custom-made anti-Epac antibodies recognize a single protein band in sperm extracts; the signal is specific because it is abolished by preincubation with the synthetic peptide against which the antibodies were raised [8]. The onset of the AR elicited by calcium in SLO-permeabilized human sperm induced Rap (Figure 2A) and Rab3 (Figure 2B) activation. Rap-GTP localized in the acrosomal region of 7.8 ± 2.2% of cells before and 29.6 ± 2.6% of cells after stimulating with calcium (Figure 2A). These results indicate that Rap is activated in the acrosomal region upon initiation of the exocytotic cascade in human sperm. They also agree with previous findings that Ral-GDS-RBD pulls down more Rap1-GTP from sperm challenged to undergo exocytosis than from untreated controls [12,17]. Likewise, Rab3-GTP localized to the acrosomal region in 9.6 ± 1.4% of cells before and 24.05 ± 3.5% of cells after stimulating with calcium (Figure 2B). Preloading of sperm with 6.7 nM anti-Epac antibodies precluded the activation of Rap (11.95 ± 3.5% cells with acrosomal Rap-GTP, Figure 2A) but not that of Rab3 (30.6 ± 1.8 cells with acrosomal Rab3-GTP, Figure 2B). These results formally demonstrate our prediction that Rab3 was active in sperm treated with the low anti-Epac antibody concentration; this is why the limb headed by Rab3-GTP was functional and the AR rescued by adenophostin A (presumably because by promoting internal calcium release, this IP_3_-mimetic reached the end point of the calcium limb pharmacologically [12]). In contrast, extracellular calcium was unable to activate Rab3 when sperm had been previously exposed to 134 nM anti-Epac antibodies (10.14 ± 3 cells with acrosomal Rab3-GTP, Figure 2B). Thus, we interpret that higher anti-Epac antibodies concentrations irreversibly blocked the AR because neither Rap nor Rab3 could bind GTP. In this context, adenophostin A could not reverse the inhibition [12] because both limbs — not only the intracellular calcium mobilization one which adenophostin A can bypass — were non-operational. Later in this manuscript we will provide direct evidence that adenophostin A provokes the emptying of the acrosomal calcium store.

Because both the anti-Epac antibodies and the anti-GST antibodies used for detection of the activity probes are raised in rabbits, we needed to make sure that the secondary antibodies detected only the anti-GST under the experimental conditions we used. An additional figure file shows that SLO-permeabilized sperm treated with 134 nM anti-Epac antibodies and calcium and processed for Rap-GTP far-immunofluorescence but without the anti-GST antibodies did not show acrosomal staining (Additional file 2). Samples run in parallel and probed with Ral-GDS-RBD followed by anti-GST and Cy3-anti-rabbit antibodies did. These controls indicate that the fluorescently labelled secondary antibodies reacted with the anti-GST but not with the anti-Epac antibodies introduced into sperm to perturb the exocytotic machinery. In other words, they demonstrate that our assay faithfully reports the activation status of sperm Rap.

Next, we asked what are the mechanisms through which anti-small GTPases antibodies halt the AR. We run far-immunofluorescence experiments to detect active Rap in SLO-permeabilized sperm previously loaded with anti-Rap1 antibodies. The specificity control for these antibodies is shown in [12]. Figure 2C shows that the percentage of sperm with Rap-GTP in the acrosomal region was comparable to that in the untreated control and approximately four fold lower than in cells challenged with calcium but without anti-Rap1 antibodies. Although raised in rabbits, the anti-Rap1 antibodies were not detected by the fluorescent secondary antibodies under the conditions used (Additional file 2). Thus, we conclude that anti-Rap1 antibodies impaired the activation of Rap1 elicited by the AR trigger.

Likewise, SLO-permeabilized sperm loaded with anti-Rab3A antibodies and challenged with calcium did not exhibit acrosomal Rab3-GTP (Figure 2D). The specificity of the effect of the anti-Rab3A antibodies was tested by blocking their active sites with recombinant Rab3A [8,16]. These results suggest that anti-Rab3A antibodies inhibit the AR because they prevent activation of endogenous Rab3.

### Reversible pairs of stage-specific blockers and their rescuers are powerful tools to investigate signaling pathways

It is relatively straightforward to analyze the sequence of events that drive each arm of the signaling pathway individually. In this paper, we tackled the far more complicated problem of scrutinizing both limbs simultaneously. Furthermore, we investigated what happens to one branch when the other is made temporarily unavailable. Luckily, we were able to exploit a remarkable feature of our permeabilized sperm model that confers the ability of reversibly halting calcium-triggered exocytosis at specific stages (with reagents that we will refer to as “inhibitor 1”). We can subsequently probe the system with a second inhibitor (“inhibitor X”) and finally rescue the initial exocytotic block with reagents that reverse the effect of inhibitor 1 (“inhibitor 1 rescue”). In short, the strategy of adding inhibitor 1 → calcium → inhibitor X → inhibitor 1 rescue in a sequence allows us to map the point when the target of inhibitor X operates in relation to inhibitor 1. The premiss we work on is that if the target of inhibitor X is only required at an early step, the reaction would not be inhibited by the late (after inhibitor 1 and calcium) addition of inhibitor X. On the contrary, if the target is required during a late phase, inhibitor X will arrest the reaction in a manner that the inhibitor 1 rescue will not be able to overcome.

Here we use three reversible pairs to test predictions that arise from our model and to gain insights into if and how do both branches of the signaling pathway connect to achieve the AR. The way these reversible pairs work is by competition, not reconstitution [16]. Briefly, inhibitor 1 binds its intracellular target, for instance Rab27 in the first pair we will describe; the reaction reaches an equilibrium between the free components and the dimer “sperm Rab27-anti-Rab27 antibody” complex, with prevalence of the latter at the antibody concentration used. Under these conditions, calcium fails to achieve the AR because Rab27 is kept unavailable. Recombinant Rab27A (neither geranylgeranylated nor GTP-loaded and therefore inactive *per se*) added subsequently to sperm displaces the antibody from the endogenous protein. This is because the mass of the recombinant protein is much larger than that of the endogenous one; in other words, the probability of the antibody encountering a recombinant molecule is much higher than that of encountering endogenous Rab27. Thus, a new equilibrium is reached between the antibody and recombinant Rab27A, releasing the endogenous protein that is now free to act. Because the system had already been exposed to calcium, the signaling cascade had achieved all the stages prior to that when Rab27 was required by the time we added the recombinant protein. We predict that if the target of inhibitor X is situated upstream of Rab27, inhibitor X — added after anti-Rab27 and calcium — would not prevent exocytosis. On the other hand, if its target is situated downstream of Rab27 on the same branch, inhibitor X would block the AR. What happens when the target is situated on the opposite branch is not so easy to anticipate and constitutes one of the goals of this study.

### Sequestration of sperm Rab27 prevents subsequent cAMP/Epac-dependent calcium mobilization from the acrosome: results obtained with the reversible pair anti-Rab27 antibody (inhibitor 1)/recombinant Rab27A (inhibitor 1 rescue)

**Figure 3:**
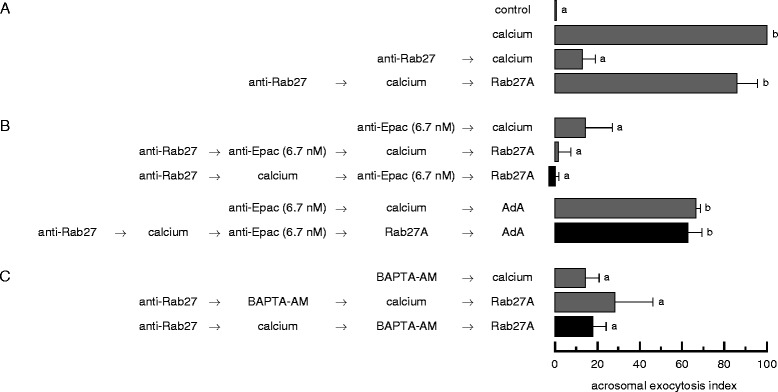
**Sequestration of sperm Rab27 prevents subsequent calcium mobilization from the acrosome: results obtained with the reversible pair anti-Rab27 antibody (inhibitor 1)/recombinant Rab27A (inhibitor 1 rescue).** SLO-permeabilized spermatozoa were loaded with 7 nM anti-Rab27 antibodies for 8 min at 37°C to block the signaling pathway where Rab27 is required. The AR was subsequently initiated by adding 0.5 mM CaCl_2_. After 8 min incubation at 37°C to allow exocytosis to proceed to the Rab27-sensitive step, sperm were treated with 6.7 nM anti-Epac antibodies **(B)** or 10 μM BAPTA-AM **(C)** and incubated for an additional 8 min at 37°C. Last, we added 14 nM recombinant GST-Rab27A (unprenylated and not loaded with nucleotides) to rescue the anti-Rab27 antibody block and incubated as before (black bars). When indicated, 5 μM adenophostin A (AdA) was added at the end of the series and samples were incubated for an additional 8 min. We run several controls in parallel (grey bars): background AR in the absence of any stimulation (control), AR stimulated by 0.5 mM CaCl_2_ (calcium), inhibition by 7 nM anti-Rab27 antibodies , 6.7 nM anti-Epac antibodies, and 10 μM BAPTA-AM; rescue of the anti-Rab27 antibodies by Rab27A; rescue of the anti-Epac antibodies by adenophostin A; and the inhibitory effect of the blockers when present throughout the experiment (anti-Rab27 → calcium → anti-Epac/BAPTA-AM → Rab27A). Sperm were fixed and AR was measured by FITC-PSA binding as described under Methods. The data represent the mean ± SEM of at least three independent experiments. Different letters indicate statistical significance (P < 0.001).

The first reversible pair we used was the anti-Rab27 antibody/recombinant Rab27A, where the antibody sequesters endogenous Rab27 and impairs its actions. The specificity controls for the anti-Rab27 antibody used in this study are shown in [16]. The recombinant protein (which is inactive because it is neither geranylgeranylated nor loaded with GTP) added at the end of the incubation displaces the antibody from sperm Rab27 and allows the exocytotic cascade to resume ([16] and Figure 3A). We used the following sequence of reagents’ addition to investigate whether Epac is required before or after Rab27: anti-Rab27 antibodies → calcium → anti-Epac antibodies (6.7 nM, inhibitor X) → recombinant Rab27A. Anti-Epac antibodies inhibited the AR under these experimental conditions (Figure 3B, top black bar). Adenophostin A rescued the anti-Epac block both in the absence ([12] and Figure 3B) and in the presence of the anti-Rab27 antibodies (Figure 3B, bottom black bar). Taken together, these results indicate that when the AR was initiated by calcium, Epac was still necessary after Rab27. We conclude that Epac’s role under these experimental settings appears to be limited to (ultimately) mobilizing intracellular calcium, which is in the other arm of the pathway compared to Rab27 (Figure 1). This is in agreement with the observation that at 6.7 nM, anti-Epac antibodies interfere with the activation of Rap but not with that of Rab3 (Figure 2A-B).

BAPTA-AM is a permeant chelating agent that accumulates in membrane-bound compartments. BAPTA-AM blocks the AR in SLO-permeabilized sperm presumably because it chelates calcium in the lumen of the acrosome and therefore prevents its mobilization to the cytosol ([11] and Figure 3C). When we used BAPTA-AM as inhibitor X, recombinant Rab27 failed to rescue the AR (Figure 3C, black bar). Thus, we conclude that keeping Rab27 unavailable affects the calcium mobilization branch of the pathway. The opposite is not true: an AR inducer can still accomplish the stage at which Rab27 is activated and required when we keep intracellular calcium unavailable with 2-aminoethoxy-diphenylborate (2-APB, an IP_3_-sensitive calcium channel blocker) or the photosensitive calcium chelator *O*-nitrophenyl EGTA-acetoxymethyl ester (NP-EGTA-AM) [16].

### Sequestration of sperm Rab3 prevents the subsequent function of NSF and calcium mobilization from the acrosome: results obtained with the reversible pair anti-Rab3A antibody (inhibitor 1)/recombinant Rab3A (inhibitor 1 rescue)

**Figure 4:**
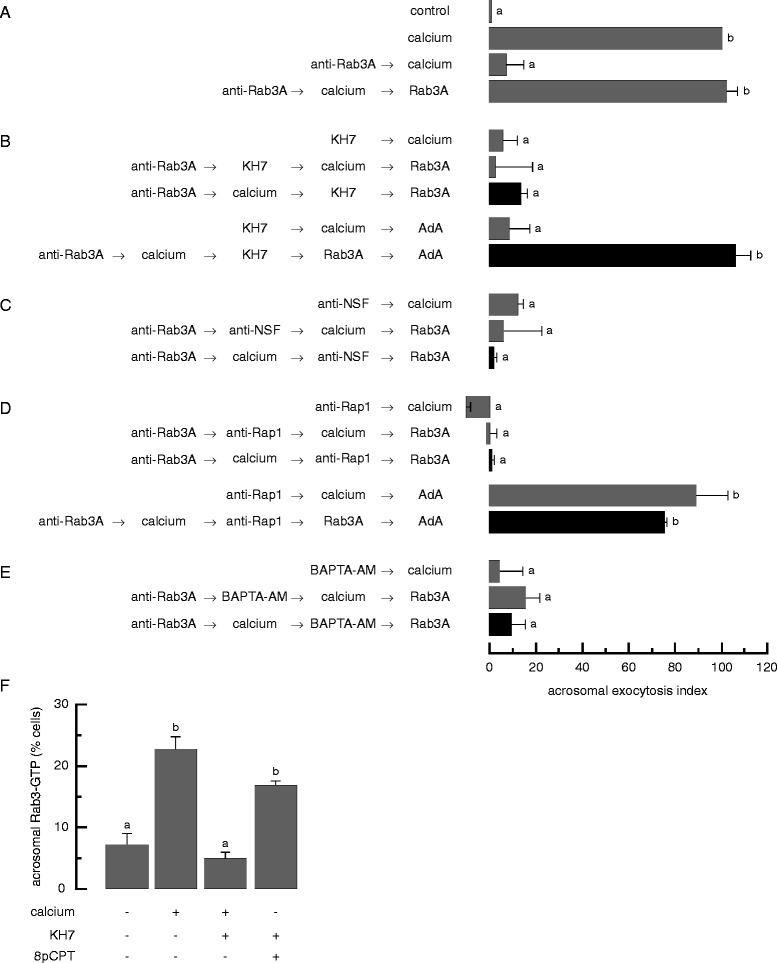
**Sequestration of sperm Rab3 prevents the subsequent function of NSF and calcium mobilization from the acrosome: results obtained with the reversible pair anti-Rab3A antibody (inhibitor 1)/recombinant Rab3A (inhibitor 1 rescue). (A-E)** SLO-permeabilized spermatozoa were loaded with 70 nM anti-Rab3A antibody. The AR was subsequently initiated with 0.5 mM CaCl_2_. Next. sperm were treated with 10 μM KH7 **(B)**, anti-NSF antibodies (whole rabbit serum diluted 1:300) **(C)**, 134 nM anti-Rap1 antibodies **(D)**, or 10 μM BAPTA-AM **(E).** Last, we added 140 nM GST-Rab3A (unprenylated and not loaded with nucleotides) to rescue the anti-Rab3A antibody block and incubated as before (black bars). When indicated, 5 μM adenophostin A (AdA) was added at the end of the series. Samples were incubated for 8 min at 37°C after each addition. We run several controls in parallel (grey bars): background AR (control), AR stimulated by 0.5 mM CaCl_2_ (calcium); inhibition by anti-Rab3A, anti-NSF, and anti-Rap1 antibodies, and BAPTA-AM; rescue of the anti-Rab3A antibody by Rab3A and of anti-Rap1 antibodies by adenophostin A; lack of rescue of KH7 by adenoposthin A; and the inhibitory effect of the blockers when present throughout the experiment (anti-Rab3A → calcium → KH7/anti-NSF/anti-Rap1/BAPTA-AM → Rab3A). Sperm were fixed and AR was measured as described. The data represent the mean ± SEM of at least three independent experiments. **(F)** SLO-permeabilized sperm were treated with 100 μM 2-APB and 10 μM KH7 before initiating the AR with 0.5 mM CaCl_2_ or 50 μM 8-pCPT-2'-O-Me-cAMP (8pCPT). Incubations were for 15 min at 37°C after each addition. Samples were processed for Rab3-GTP immunodetection as described. Shown is the percentage of cells immunodecorated in the acrosomal region with the anti-GST antibodies. The data represent the mean ± SEM of at least three independent experiments. Different letters indicate statistical significance (P < 0.001).

The second reversible pair we used, anti-Rab3A antibodies/non-geranylgeranylated, recombinant Rab3A (Figure 4A), is based on the same principles described above for the Rab27-related pair. NSF is situated on the same arm as Rab3 (Figure 1). When anti-NSF antibodies were used as inhibitor X, the AR was still inhibited after addition of recombinant Rab3A (inhibitor 1 rescue) (Figure 4C). In other words, in the absence of Rab3A, fusion remained sensitive to anti-NSF antibodies (please see [8] for specificity controls). These experiments lend support to our model for the AR, where endogenous NSF is required after sperm Rab3 during the exocytotic cascade ([12] and Figure 1).

Data shown in Figure 3 indicate that blocking the fusion machinery arm at the stage when Rab27 is required halted the calcium mobilization arm of the pathway. Therefore we asked what happens if we interfere with the fusion machinery arm downstream of Rab27 with anti-Rab3A antibodies as inhibitor 1. BAPTA-AM used as inhibitor X prevented the AR (Figure 4E, black bar). These results suggest that the AR inducer was not able to accomplish intracellular calcium mobilization when the activation (Figure 2D) and functions of sperm Rab3A were impaired by the anti-Rab3A antibodies. In the last part of this manuscript we present direct evidence that substantiates this premiss. Furthermore, these data strengthen the observations made with the anti-Rab27/Rab27A reversible pair, that the calcium mobilization arm needs the protein machinery arm to succeed. Once again, the opposite is not true because experiments conducted with NP-EGTA-AM and anti-Rab3A antibodies suggest that the protein machinery arm does not need the calcium mobilization arm [18]. Furthermore, Rab3 exchanges GDP for GTP in response to exocytotic inducers even when the internal store is kept unavailable with 2-APB (Figures 2B, 4F and [16]).

In an attempt to narrow down at what level do the pathways crosstalk, we went “up” in the calcium mobilization limb and tested the effect of anti-Rap1 antibodies (inhibitor X) in combination with the pair anti-Rab3A/Rab3A. In the absence of active Rab3, the system was susceptible to this Rap blocker (Figure 4D, top black bar). These findings led us to conclude that Rap-GTP (because the anti-Rap1 antibodies prevent the activation of Rap, Figure 2C) exerted at least some of its functions downstream those of Rab3 (Figure 1).

Our model for the AR cascades situates sAC upstream of Rab3 (Figure 1). Therefore, we tested by far-immunofluorescence the hypothesis that KH7 should prevent the activation of sperm Rab3 elicited by calcium and found that this was indeed the case (Figure 4F). We tested the effect of KH7 in sperm challenged with 8-pCPT-2'-O-Me-cAMP as a control. This Epac analogue bypasses the requirement of sAC and cAMP synthesis in AR assays [12] and directly or indirectly activates sperm as well as exogenous Rab3 as reported by pull down assays [12,16]. The results in Figure 4F show that 8-pCPT-2'-O-Me-cAMP increases the population of sperm with acrosomal Rab3-GTP measured by far-immunofluorescence and that this effect is insensitive to KH7.

Surprisingly, KH7 as inhibitor X prevented the calcium-triggered AR when added after loading sperm with anti-Rab3A antibodies (Figure 4B, top black bar). We had not anticipated these results because in our model sAC is situated upstream of Rab3. How could this be? Once again we resorted to adenophostin A and found that it rescued the inhibitory effects of both anti-Rap1 antibodies (Figure 4D, bottom black bar), and KH7 (Figure 4B, bottom black bar). These data indicate that continuous synthesis of cAMP by sAC is required to mobilize intracellular calcium when the AR is halted by a Rab3A blocker. In the last section of this manuscript we show that cAMP/Epac are mandatory for the acrosomal calcium mobilization elicited by external calcium. Taken together, the data generated with anti-Rab27/Rab27A and anti-Rab3A/Rab3A pairs indicate that the calcium mobilization arm is not independent of the fusion machinery arm; whatever the crosstalk, the former cannot happen as long as secretory Rabs are kept unavailable (Figure 1). The opposite is not true: Rab3 exchanges GDP for GTP normally in the absence of Rap [12] and in the presence of 2-APB (Figures 2B, 4F and [16]).

### Inhibition of sperm PTP1B does not interfere with intracellular calcium mobilization from the acrosome: results obtained with the reversible pair substrate trapping mutant PTP1BD181A (inhibitor 1)/wild type PTP1B (rescue inhibitor 1)

**Figure 5:**
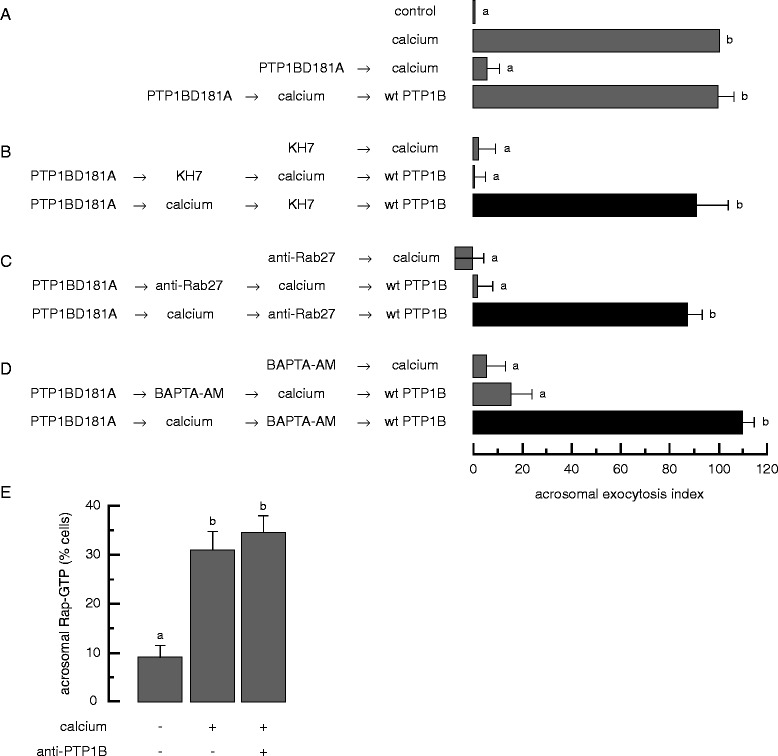
**Inhibition of sperm PTP1B does not interfere with intracellular calcium mobilization from the acrosome: results obtained with the reversible pair substrate trapping mutant PTP1BD181A (inhibitor 1)/wild type PTP1B (rescue inhibitor 1).** SLO-permeabilized spermatozoa were loaded with 300 nM PTP1BD181A for 8 min at 37°C to block the signaling pathway where PTP1B is required. The AR was subsequently initiated by adding 0.5 mM CaCl_2_. After 8 min incubation at 37°C to allow exocytosis to proceed to the PTP1B-sensitive step, sperm were treated with 10 μM KH7 **(B)**, 7 nM anti-Rab27 antibodies **(C)**, or 10 μM BAPTA-AM **(D)**, and incubated for an additional 8 min at 37°C. Last, we added 27 nM wild type PTP1B (wt PTP1B) to rescue the mutant PTP1B block and incubated as before (black bars). We run several controls in parallel (grey bars): background AR in the absence of any stimulation (control), AR stimulated by 0.5 mM CaCl_2_ (calcium); inhibition by 10 μM KH7, 7 nM anti-Rab27 antibodies, and 10 μM BAPTA-AM; rescue of PTP1BD181A by wild type PTP1B; and the inhibitory effect of the blockers when present throughout the experiment (PTP1BD181A → calcium → KH7/anti-Rab27/BAPTA-AM → wt PTP1B). Sperm were fixed and AR was measured by FITC-PSA binding as described under Methods. The data represent the mean ± SEM of at least three independent experiments. **(E)** SLO-permeabilized sperm were treated with 3.3 nM anti-PTP1B antibodies. The AR was initiated with 0.5 mM CaCl_2_. Incubations were for 15 min at 37°C after each addition. Samples were processed for Rap-GTP immunodetection as described under Methods. Shown is the percentage of cells immunodecorated in the acrosomal region with the anti-GST antibodies, representing active Rap. The data represent the mean ± SEM of at least three independent experiments. Different letters indicate statistical significance (P < 0.001).

All the data gathered with anti-Rab3A and anti-Rab27 antibodies as AR blockers indicate that the protein machinery arm is necessary to mobilize intracellular calcium, the final event in the other arm of the pathway (Figure 1). How far downstream of Rab3 can the system advance until it reaches a stage in which the protein arm no longer affects the other? We know this happens eventually, because calcium can be mobilized from the acrosome in the presence of botulinum toxin E. This protease hydrolyzes SNAP-25 and thus blocks a very late stage in the protein arm of the cascade [9,18]. In sperm undergoing exocytosis, PTP1B is necessary to dephosphorylate NSF and thus disassemble fusion-incompetent *cis* SNARE complexes (the predominant configuration in resting sperm) into reactive monomeric syntaxin1, synaptobrevin2 and SNAP-25 [19], which are toxin-sensitive. We showed in Figure 4C that NSF is required after Rab3 and inferred that PTP1B is also situated downstream of Rab3. To answer the question posed before about how far after Rab3 can we go before the two signaling pathways become independent we forced the protein machinery limb to move downstream of Rab3 with the reversible pair substrate trapping mutant PTP1BD181A/wild type PTP1B (Figure 5A). Our model predicts that early stages should have been accomplished before we blocked PTP1B’s activity. Results depicted in Figure 5B (black bar) show that KH7 failed to inhibit the AR when added after PTP1BD181A and calcium. These data suggest that calcium had accomplished cAMP synthesis normally, and this had been used by the time we added KH7 to the cells, regardless of the presence of PTP1BD181A in the system. Likewise, when we applied the anti-Rab27 antibody as inhibitor X instead of KH7 the AR proceeded normally (Figure 5C, black bar). These results agree with the hypothesis that within the context of this reversible pair, the AR is insensitive to blockers whose targets are required before PTP1B (Figure 1).

Next, we investigated whether or not preventing phospho-NSF tyrosine dephosphorylation with PTP1BD181A affected the other branch of the pathway. We could not anticipate the results because we knew that blocking Rab3 affected the other branch but blocking SNARE complex assembly did not (Rab3 is upstream of PTP1B and SNARE complex assembly is downstream of this phosphatase, all three are in the protein machinery limb, Figure 1). When we chelated calcium in the lumen of the acrosome with BAPTA-AM, we observed no inhibition of the AR (Figure 5D, black bar). This result suggests that maintaining the protein machinery arm artificially off with PTP1BD181A made the system refractory to an intracellular calcium mobilization blocker. These data hint toward the independence of the intra-acrosomal calcium release arm from the membrane fusion machinery arm of the pathway as long as the latter is halted at or after the PTP1BD181A-sensitive step. The simplest explanation for the observations made with PTP1BD181A as inhibitor 1 is that the AR trigger had mobilized intracellular calcium before BAPTA-AM have had the opportunity to prevent it. Results described in the last part of this manuscript demonstrate that incubation with PTP1BD181A does not perturb rapid calcium mobilization from the acrosomal reservoir.

It would appear that the end point of the intra-acrosomal calcium release arm of the pathway is independent of the protein machinery arm when the system is allowed to advance up to the point when PTP1B is required. Intracellular calcium mobilization happens downstream of Rap activation, we next asked whether maintaining PTP1B unavailable would impede the extracellular calcium-triggered activation of Rap. We chose to use an anti-PTP1B antibody instead of the mutant GST-PTP1BD181A to avoid detection problems when probing the slides with the anti-GST antibodies for immunofluorescence (see [19] for the anti-PTP1B antibody specificity control). Similar percentages of cells stimulated with calcium exhibited acrosomal Rap-GTP staining regardless of the pretreatment with anti-PTP1B antibodies (Figure 5E). In short, sequestration of PTP1B did not have a detrimental effect on the activation of Rap, thus confirming the independence of the pathways after the PTP1B-sensitive step.

In summary, when we halted the AR with PTP1BD181A as inhibitor 1, we found that the calcium mobilization arm, including the activation of Rap, was independent of the fusion machinery arm, whereas when we applied anti-Rab3 as inhibitor 1, the calcium mobilization arm depended on the integrity of the fusion machinery arm. This means that the connection between pathways is located somewhere downstream of Rab3 and upstream of PTP1B (Figure 1).

### As AR inducer, extracellular calcium mobilizes calcium from the acrosome in a cAMP-, Rab3- and IP_3_-dependent but PTP1B-independent fashion

**Figure 6:**
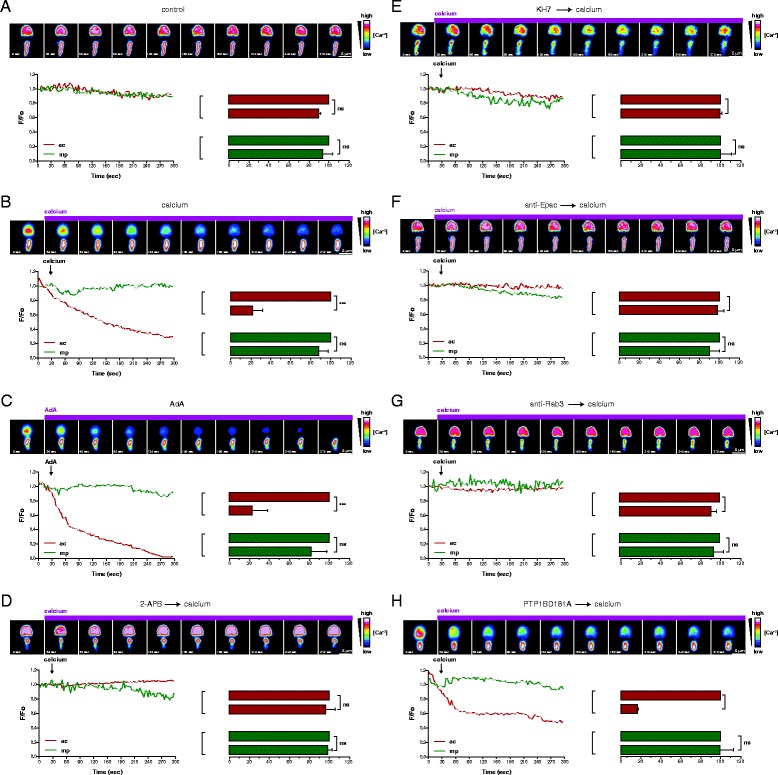
**Extracellular calcium elicits calcium release from the acrosomal store through IP**_**3**_ **-gated channels in a cAMP/Epac and Rab3-dependent manner.** SLO-permeabilized human sperm were treated with 100 nM botulinum toxin B to prevent exocytosis, loaded with the fluorescent calcium indicator Fluo3-AM (2 μM) and incubated with 100 μM 2-APB **(D)**, 10 μM KH7 **(E)**, 6.7 nM anti-Epac **(F)** or 70 nM anti-Rab3A **(G)** antibodies, 300 nM PTP1BD181A **(H)**. Samples were stimulated with 0.5 mM CaCl_2_ (calcium, **B**, **D-H**) or 5 μM adenophostin A (AdA, **C**) and the fluorescence intensity visualized as described in the Methods section. Images at top are pseudo-coloured to show intensity of fluorescence. Scale is shown on right (“warm” colours show high [Ca^2+^]). Changes in fluorescence are localised to the acrosome; fluorescence in the mid piece was invariant. Scale bars = 5 μm. Each line graph shows the recording of [Ca^2+^] changes in the cell shown above. Plotted are the normalized Fluo3 fluorescence variations in the acrosome (ac, red) and the mid piece (mp, green) in response to the application of CaCl_2_ or adenophostin A (indicated by vertical arrows) vs. time. Bar graphs illustrate the population response to each treatment (mean ± SEM; 17–50 cells in three experiments). Bar graphs show the relative fluorescence from the acrosomal (ac, red) and mid piece (mp, green) regions at the beginning (0 sec, assigned 100%) and 300 seconds after the initiation of the recording. Asterisks indicate significant difference (**P < 0.01, ***P < 0.001) from the initial value (single group analysis, 99.9% confidence interval), ns indicates that statistical differences between bars were non-significant (P > 0.05). Note: around 80% cells responded to CaCl_2_ in all experiments included in the analysis.

We have shown the influence of anti-Epac (Figure 3B) and anti-Rap1 (Figure 4D) antibodies, KH7 (Figures 4B and 5B) and their rescue — or lack of — by adenophostin A, and BAPTA-AM (Figures 3C, 4E, and 5D) on the AR in the context of three reversible pairs. Based on the results we obtained, we deduced whether they affected or not intra-acrosomal calcium mobilization. Next, we tested the veracity of these predictions by single-cell confocal microscopy. Fluo3-AM is a fluorescence indicator of intracellular calcium that is practically non-fluorescent in its ligand-free form. When sperm with their plasma membrane permeabilized with SLO are exposed to Fluo3-AM, the dye diffuses into the acrosome, where esterases remove the AM moiety, trapping the sensor in this compartment. Once inside the acrosome, Fluo3 becomes fluorescent upon binding to calcium. Thanks to the SLO permeabilization protocol, we could actually visualize the human sperm acrosomal calcium store with Fluo3-AM (Figure 6A and [9,11]), something not achievable in non-permeabilized sperm, where cytosolic esterases remove the AM moiety, trapping membrane impermeant Fluo3 in the cytosol. The concentration of calcium in the granule remained high and without appreciable changes in concentration during the 5 min incubation at 37°C (Figure 6A). This does not mean that the pool of calcium in this reservoir is static. We know from previous experiments conducted with the inhibitors cyclopiazonic acid and thapsigargin that calcium passively diffuses out from the acrosome and is pumped back into it by calcium-ATPases [11].

Next, we wanted to establish whether or not it was technically possible to measure the depletion of this store elicited by extracellular calcium, the AR inducer we used throughout the paper, without it masking the acrosomal fluorescent signal. That extracellular calcium promotes intracellular calcium mobilization is one of the theoretical foundations on which our model is built, but up until now it had not been formally demonstrated. The data summarized in Figure 6B show that this measurement is indeed feasible. To avoid confusion in the read out between calcium signal loss (due to efflux) and acrosomal loss (due to exocytosis), we performed these measurements in the presence of botulinum toxin B, a late AR blocker [18]. The high intra-acrosomal calcium concentrations typical of resting cells decreased appreciably during the first 30 sec incubation with external calcium and dropped to almost undetectable levels after 120 seconds. The mid piece harbours the sperm mitochondria; as expected, calcium from this reservoir was not mobilized in response to the onset of exocytosis (Figure 6, green lines and bars).

We and others had proposed that intra-acrosomal calcium mobilization is gated through IP_3_-sensitive channels. We provide here two pieces of direct evidence in support of this view. First, the IP_3_-mimetic drug adenophostin A provoked a rapid emptying of the acrosomal calcium reservoir (Figure 6C). Second, the IP_3_-channel blocker 2-APB prevented intracellular mobilization in response to extracellular calcium (Figure 6D).

Last, we assessed directly the influence of four reagents that halt the AR at different stages, two before (KH7 and anti-Epac antibodies) and two after the bifurcation. From the latter we selected one that acts before (anti-Rab3 antibodies) and one after (PTP1BD181A) the point in which functional data suggest that the two limbs of the pathway connect (Figure 1). sAC and Epac are required early on the cascade and functional data suggest their requirement for intra-acrosomal calcium mobilization. Accordingly, when these enzymes were kept inactive/unavailable with KH7 (Figure 6E) or anti-Epac antibodies (Figure 6F), external calcium failed to induce vesicular calcium efflux. These results provide direct evidence that cAMP and its target Epac are essential to mobilize intra-acrosomal calcium. Because PTP1BD181A acts on the fusion machinery arm after the point of crosstalk, we predicted that it would not prevent external calcium-induced-acrosomal calcium mobilization. This was indeed what we found: sperm preincubated with the mutant phosphatase mobilized intracellular calcium normally (Figure 6H). Because the anti-Rab3 antibodies halt the fusion machinery arm at or before the point of crosstalk, we hypothesized that they should interfere with acrosomal calcium mobilization. Accordingly, preincubation of sperm with these antibodies prevented external calcium-induced-acrosomal calcium mobilization (Figure 6G). These results are in agreement with those obtained with the reversible pairs PTP1BD181A/wild type PTP1B (Figure 5D) and anti-Rab3/Rab3A (Figure 4E) in combination with BAPTA-AM. Taken together, our results indicate that there is a connection between the calcium mobilization and protein machinery arms downstream of Rab3 and upstream of PTP1B (Figure 1).

## Conclusions

Our findings provide direct evidence for the existence of critical pathways underlining the AR and their crosstalk. We have taken advantage of the fact that signaling pathways during the AR can be reversibly interrupted and resumed at defined stages. The results we present here, obtained with three different strategies and a number of specific tools, were entirely consistent with each other. Furthermore, they showed that all the predictions derived from our molecular model for sperm exocytosis were correct; thus, we grew confident in the robustness of this model (summarized in Figure 1). If the AR cascade initiated by an increase of cytosolic calcium from an external source is perturbed at the point when Rab27 or Rab3 must be activated, the system halts and does not achieve intracellular calcium efflux. On the contrary, if the cascade is allowed to advance further and reach the stage when PTP1B is required, it will inevitably evolve to accomplish acrosomal calcium efflux. Thus, one conclusion derived from our findings is that the Rab3-driven and Rap-driven arms of the exocytotic cascade are joined at or near the stage catalyzed by Rab3 in a unidirectional, hierarchical connection in which the intra-acrosomal calcium mobilization arm is subordinated to the fusion protein arm; after Rab3, the pathways become independent (Figure 1). It is worth to point out that when diacylglycerol is used as AR trigger (diacylglycerol is a component of the calcium-mobilization arm of the pathway), sperm Rab3 is activated [9]. These results suggest that, under certain conditions, the calcium-mobilization arm can influence the protein machinery arm of the cascade. These findings do not contradict the ones reported in this paper if we consider that the activatory effect of diacylglycerol on Rab3 is part of a positive feedback mechanism that serves to amplify the signals and is revealed when the lipid is added exogenously. We propose the existence of a loop that connects Rab3 with cAMP/Epac. All components of this loop must be available and active in order to achieve secretion. Once the system has progressed beyond the loop, the calcium mobilization limb will proceed regardless of the fusion machinery limb. We do not know at this point all the components of the loop, but we can speculate that the Rab3 effector RIM might play some part in it based on its reported interaction with Epac [20]. Importantly, RIM is present in human sperm [21]. Whichever the mechanism, we hypothesize that its purpose is to target Epac and therefore Rap-GTP to the tethering sites created by active Rab3. In this way, intravesicular calcium will be released at or near the contact points between the acrosomal and plasma membranes. This hypothetical requirement for a highly localized calcium signal explains the dependence of the AR on intracellular calcium mobilization and why this cannot be overcome with high overall cytosolic concentrations from an extracellular source. Does the resistance to BAPTA-AM when PTP1B is inhibited argue against this calcium-dependence? We find it hard to believe that there is calcium-independent membrane fusion based on all the evidence we have gathered over the years. We favor alternative explanations, for instance that calcium released in the vicinity of the acrosome is used up immediately (i.e. through binding to synaptotagmin) or it “lingers” in the cell until later use.

## Methods

### Reagents

Recombinant SLO was obtained from Dr. Bhakdi (University of Mainz, Mainz, Germany). Spermatozoa were cultured in Human Tubal Fluid media (as formulated by Irvine Scientific, Santa Ana, CA) supplemented with 0.5% bovine serum albumin (HTF media). The rabbit polyclonal anti-Rab27 (affinity purified with the immunogen) and anti-NSF (whole serum) antibodies plus the mouse monoclonal anti-Rab3A (clone 42.2, subtype IgG2b, affinity purified on protein A-Sepharose) were from Synaptic Systems (Göttingen, Germany). Mouse monoclonal anti-PTP1B (purified IgG2a, clon 15) was from BD Transduction Laboratories™ (San Jose, CA). The rabbit polyclonal anti-GST antibody (purified IgG) was from EMD Millipore Corporation (Billerica, MA). The rabbit polyclonal antibodies against Epac were generated by Genemed Synthesis, Inc. (San Francisco, CA) using the synthetic peptide LREDNCHFLRVDK, and affinity purified on immobilized Epac peptide [8]. Anti-Rap1 A/B rabbit polyclonal antibodies were raised against the peptide EDERVVGKEQGQNLC and affinity purified on immobilized peptide (GenScript Corporation, Piscataway, NJ) [12]. Cy™3-conjugated donkey anti-rabbit IgG (H + L) was from Jackson ImmunoResearch (West Grove, PA). 8-pCPT-2'-O-Me-cAMP was from Axxora, LLC (San Diego, CA). KH7 was purchased from R&D Systems (Minneapolis, MN). Adenophostin A hexasodium salt, and 2-APB from Calbiochem were purchased from Merck Química Argentina S.A.I.C. (Buenos Aires, Argentina). 1-[2-Amino-5-(2,7-dichloro-6-hydroxy-3-oxo-9-xanthenyl)phenoxy]-2-(2-amino-5-methylphenoxy) ethane-N,N,N',N'-tetraacetic acid, pentaacetoxymethyl ester (Fluo3-AM, FluoroPure™ grade) was from Life Technologies (Buenos Aires, Argentina). Glutathione-Sepharose and Ni-NTA-agarose were from GE Healthcare (Buenos Aires, Argentina). All other chemicals were purchased from Sigma-Aldrich™ Argentina S.A., Genbiotech, or Tecnolab (Buenos Aires, Argentina).

### Recombinant proteins

A pGEX-6p (GE Healthcare) construct encoding Rab27A was a kind gift from Dr. D. Munafó (The Scripps Research Institute, La Jolla, CA). Plasmid pGEX-2T containing the cDNA-encoding human Rab3A was provided by Dr. P. Stahl (Washington University, St. Louis, MO). GST fusion proteins with the Rab3-GTP-binding domain of rat RIM (amino acids 11–398, RIM-RBD) [22] in a pGEX2p vector was generously provided by Dr. R. Regazzi (University of Lausanne, Lausanne, Switzerland). The Rap1-GTP binding cassette Ral-GDS-RBD fused to GST [23] was a kind gift from Dr. O. Coso (Universidad de Buenos Aires, Buenos Aires, Argentina). Two expression plasmids encoding amino acids 1–321 of PTP1B were kindly provided by Dr. N. Tonks: the substrate-trapping mutant PTP1B D181A fused to GST was in pGEX-3X (GE Healthcare) and the wild type PTP1B fused to His_6_ was in pET21b (Stratagene, La Jolla, CA). The light chain of botulinum toxin B fused to His_6_ (pQE3, Qiagen) was generously provided by Dr. T. Binz (Medizinische Hochschule Hannover, Hannover, Germany).

His_6_-PTP1B wild type was expressed in *E.coli* BLR(DE3) (Stratagene) by inducing with 0.5 mM isopropyl-β-D-thio-galactoside (IPTG) overnight at 22°C. The plasmid construct encoding botulinum toxin B was transformed into *E.coli* M15pRep4 (Qiagen) and protein expression was induced 4 h at 30°C with 1 mM IPTG. Plasmids encoding all GST-fused proteins were transformed in *E.coli* BL21(DE3) T1^R^ and protein expression was induced with IPTG as follows: GST-Rab3A and GST-RIM-RBD, 0.5 mM IPTG for 3 h at 37°C; GST-Ral-GDS-RBD 0.1 mM IPTG, overnight at 22°C; GST-PTP1BD181A and GST-Rab27A 1 mM IPTG, 3 h at 37°C. GST-fused recombinant proteins were purified on glutathione-Sepharose beads following standard procedures. Purification of His_6_-tagged botulinum toxin B was was carried out under native conditions according to Qiagen’s instructions. Purification of wild type PTP1B was also carried out under native conditions according to Qiagen’s instructions except that the purification buffers contained 20 mM TrisHCl, pH 7.4 instead of 50 mM phosphate, pH 8; NaCl was 300 mM; lysis buffer contained 8–10 mM imidazole, washing buffer contained 20 mM imidazole; and elution buffer contained 250 mM imidazole.

### Human sperm sample preparation procedure. AR assay

Human semen samples were obtained from normal healthy donors. Semen was allowed to liquefy for 30–60 min at 37°C. Following a swim-up protocol to isolate highly motile cells, sperm concentrations were adjusted to 7 x 10^6^/ml before incubating for at least 2 h under capacitating conditions (HTF, 37°C, 5% CO_2_/95% air). Sperm were washed twice with PBS and resuspended in cold PBS containing 7.5 U/ml SLO for 15 min at 4°C. Cells were washed once with PBS and resuspended in ice-cold sucrose buffer (250 mM sucrose, 0.5 mM EGTA, 20 mM HEPES-K, pH 7) containing 2 mM DTT. We added inhibitors and 0.5 mM CaCl_2_ sequentially as indicated in the figure legends and incubated for 8–15 min at 37°C after each addition. Sperm were spotted on teflon-printed slides, air dried and fixed/permeabilized in ice-cold methanol for 20 sec. An alternatively fixation protocol (described under **“Detection of GTP-Rab3 and GTP-Rap by far-immunofluorescence”**) rendered identical results. Acrosomal status was evaluated by staining with FITC-coupled *Pisum sativum* agglutinin (FITC-PSA, 25 μg/ml in PBS) for 40 min at room temperature followed by a 20 min wash in water [24]. We scored at least 200 cells per condition using an upright Nikon Optiphot II microscope equipped with epifluorescence optics. Basal (“control”, no stimulation) and positive (“calcium”, 0.5 mM CaCl_2_) corresponding to 10 μM free calcium estimated by MAXCHELATOR, a series of program(s) for determining the free metal concentration in the presence of chelators; available on the World Wide Web at http://www.stanford.edu/~cpatton/maxc.html, Chris Patton, Stanford University, Stanford, CA) controls were included in all experiments. Acrosomal exocytosis indices were calculated by subtracting the number of spontaneously reacted spermatozoa (basal control without stimulation, ranged from ≈ 8 to 30% before normalization) from all values and expressing the results as a percentage of the AR observed in the positive control (ranged from ≈ 17 to 40% before normalization; assigned 100% of responsive cells for normalization). We only included in our analysis results derived from experiments that produced similar responses and where the difference between basal and calcium-stimulated conditions was of at least eight-ten percentage points. Samples with a level of spontaneously reacted sperm higher than 30% were excluded from our analysis. Data were evaluated using the Tukey-Kramer post hoc test for pairwise comparisons. Differences were considered significant at the P < 0.05 level.

### Detection of GTP-Rab3 and GTP-Rap by far-immunofluorescence

Capacitated, SLO-permeabilized sperm suspensions were incubated with or without 100 μM 2-APB for 10 min at 37°C to prevent acrosomal loss due to exocytosis, treated with AR blockers and/or inducers (10–15 min at 37°C after each addition), fixed in 2% paraformaldehyde (15 min at room temperature), and the fixative was neutralized with PBS containing 100 mM glycine (overnight at 4°C). 2–3.5 × 10^5^ cells were attached to poly-L-lysine coated, 12 mm round coverslips by incubating for 30 min at room temperature in a moisturized chamber. Sperm membranes were permeabilized in 0.1% Triton X-100 in PBS for 10 min at room temperature, cells were washed three times (6 min each) with PBS containing 0.1% polyvinylpyrrolidone (PVP, average M.W. = 40,000; PBS/PVP), and non-specific reactivity was blocked in 5% bovine serum albumin in PBS/PVP for 1 h at 37°C. Slides were overlain with 140 nM GST-RIM-RBD or GST-Ral-GDS-RBD in 5% bovine serum albumin in PBS/PVP for 1 h at 37°C. After washing (three times, 6 min each, PBS/PVP), coverslips were incubated with anti-GST antibodies (31.5 μg/ml = 210 nM, 1 h at 37°C) in a moisturized chamber. After washing three times for 6 min with PBS/PVP, we added Cy™3-conjugated donkey anti-rabbit IgG (2,5 μg/ml in 1% bovine serum albumin in PBS/PVP) and incubated for 1 h at 37°C protected from light. Coverslips were washed three times for 6 min with PBS/PVP. Cells were subsequently stained for acrosomal content as described before but without air drying, mounted with Mowiol^®^ 4–88 containing 2 μM Hoechst 33342 and stored at room temperature in the dark until examination with an Eclipse TE2000 Nikon microscope equipped with a Plan Apo 40x/1.40 oil objective and a Hamamatsu digital C4742-95 camera operated with MetaMorph 6.1 software (Universal Imaging Corp., USA). We scored the presence of immunostaining in the acrosomal region by manually counting between 100 and 200 cells either directly at the fluorescence microscope or in digital images from at least 10 fields. The Tukey-Kramer post hoc test was used for pairwise comparisons. Background was subtracted and brightness/contrast were adjusted to render an all-or nothing labeling pattern using Image J (freeware from N.I.H.).

### Single cell imaging of intra-acrosomal calcium

Capacitated, SLO-permeabilized sperm (250 μl aliquots, 4 × 10^7^ cells/ml) suspended in sucrose buffer were incubated with 100 nM botulinum toxin B (preactivated with 1 mM DTT during 15 minutes at 37°C) and then loaded with Fluo3-AM (2 μM, dispersed with pluronic acid F-127, 0.08%) at 37°C for 30 min. Cells were washed once, resuspended in sucrose buffer and treated or not for 15 min at 37°C with the AR blockers to test. Twenty-five μl of each sample were gently introduced into an imaging chamber fitted with a poly-L-lysine-coated 25 mm round coverslip. The chamber was then transferred to the microscope stage, washed with sucrose buffer to remove excess dye and unbound sperm and replenished with 300 μl fresh sucrose buffer. After the baseline was stabilized, 0.5 mM CaCl_2_ or 5 μM adenophostin A were added to the medium. Samples were maintained at 37°C at all times. A 473 nm laser with an emission of 535 nm was used to generate the excitation for Fluo3. A Plan Apo 60x objective was used for imaging on an Olympus FV 1000 confocal microscope. Images were collected in every 3 sec using the Fluoview FV-1000 software. Fluorescence measurements in individual sperm were made by manually drawing a region of interest around the acrosome and midpiece of each cell. Results are presented as pseudo color [Ca^2+^] images. Cells with uneven dye loading were excluded from the analysis. Raw intensity values were imported into Microsoft Excel and normalized using F/Fo ratios after background subtraction, where F is fluorescence intensity at time t and Fo is the baseline as calculated by averaging the 10 frames before additions. Cells with peak changes of >50% in F/Fo after application of CaCl_2_ were counted as responsive. The total series of F/Fo are plotted versus time. Relative fluorescence (%) is the fluorescence normalized to that obtained before the addition of CaCl_2_. Experiments were carried out between two and four times, each repeat on a different sample.

## Additional files

Additional file 1: Figure S1. Title of data: A novel far-immunofluorescence method to detect the localization of Rap-GTP. Description of data: supporting Figure S1 with legend: Capacitated, SLO-permeabilized sperm were incubated with 2-APB and EDTA as described under Methods. Cells were loaded with 40 μM GDP-β-S or GTP-γ-S in a buffer with low MgCl_2_ concentrations during 10 min at 37°C and bound nucleotides stabilized with 15 mM MgCl_2_ (5 min at 37°C). Cell suspensions were fixed in 2% paraformaldehyde, attached to poly-L-lysine coated coverslips, and overlain with 140 nM GST-Ral-GDS-RBD in blocking solution. Cells were triple stained with an anti-GST antibody as read out for the activity probe that detects active Rap (red, left panel), FITC-PSA (to confirm that the AR was effectively prevented by 2-APB; green, central panels), and Hoechst 33342 (to visualize all cells in the field; blue, right panels). Bars = 5 μm. Additional file 2: Figure S2. Title of data: Primary antibodies raised in rabbits and introduced into sperm through SLO-generated pores do not interfere with the anti-GST read out in far-immunofluorescence experiments. SLO-permeabilized sperm were treated initially with 100 μM 2-APB, next with 134 nM anti-Epac or anti-Rap1 antibodies as indicated in the key, and finally with 0.5 mM CaCl_2_. Incubations were for 15 min at 37°C after each addition. The top two panels correspond to samples processed for Rap-GTP immunodetection as described under Methods (red, left panel). The remaining two panels correspond to samples overlain with Ral-GDS-RBD followed by anti-rabbit Cy3 but without anti-GST antibodies in between (red, left panel). Cells were stained with FITC-PSA (to confirm that the AR was effectively prevented by 2-APB; green, central panels) and Hoechst 33342 (to visualize all cells in the field; blue, right panels). Bars = 5 μm.

## Abbreviations

2-APB: 2-aminoethoxy-diphenylborate
AR: Acrosome reaction
Epac: Exchange protein directly activated by cAMP
FITC-PSA: FITC-coupled *Pisum sativum* agglutinin
Fluo3-AM: 1-[2-Amino-5-(2,7-dichloro-6-hydroxy-3-oxo-9-xanthenyl)phenoxy]-2-(2-amino-5-methylphenoxy)ethane-N,N,N',N'-tetraacetic acid, pentaacetoxymethyl ester
GEF: Guanine-nucleotide exchange factor
GST: Gluthation-S-transferase
IP_3_: Inositol 1, 4, 5 triphosphate
IPTG: Isopropyl-β-D-thio-galactoside
NP-EGTA-AM: *O*-nitrophenyl EGTA acetoxymethyl ester
8-pCPT-2'-O-Me-cAMP: 8-(p-chlorophenylthio)-2'-O-methyladenosine-3',5'-cyclic monophosphate
PVP: Polyvinylpyrrolidone
PTP1B: Protein tyrosine phosphatase 1B
sAC: soluble adenylyl cyclase
SLO: Streptolysin O
