# Supplementary figures and images for: Epac, Rap and Rab3 act in concert to mobilize calcium from sperm’s acrosome during exocytosis
Source: Cell Commun Signal. 2014 Aug 27;12:43. doi: 10.1186/s12964-014-0043-0 (PMC4156617; doi:10.1186/s12964-014-0043-0)

GDP- $\beta$ -S

anti-GST

FITC-PSA

Hoescht

GTP- $\gamma$ -S

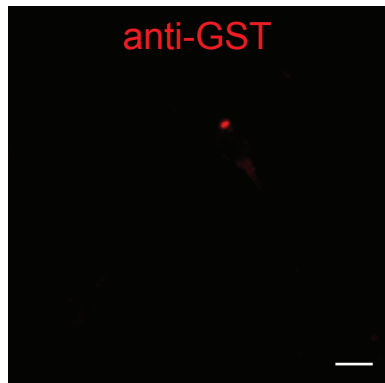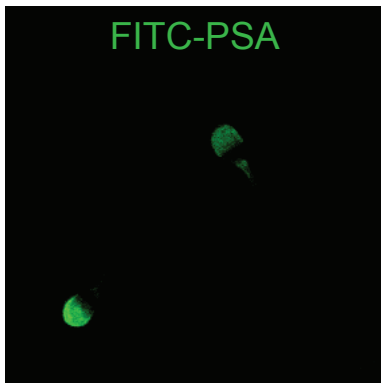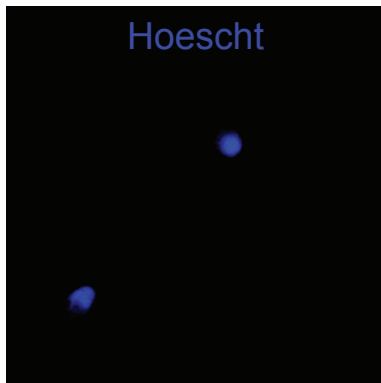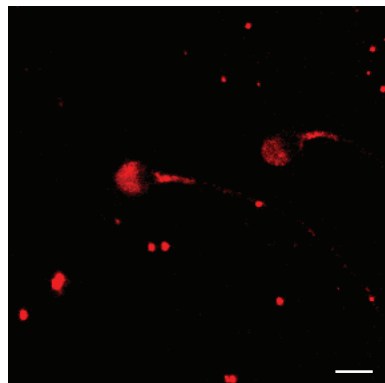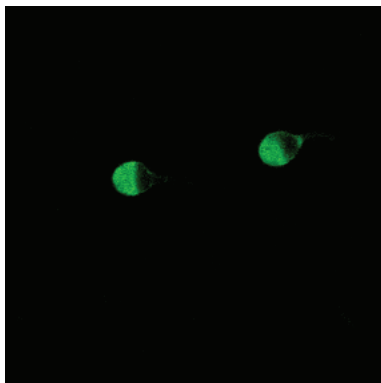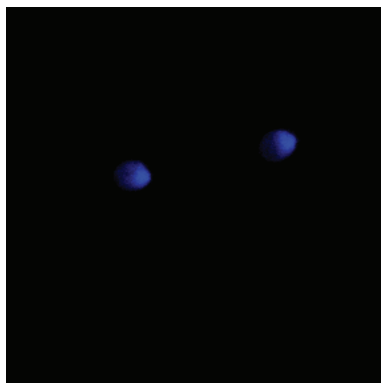

Supplement: Additional file 1: Figure S1. — Title of data: A novel far-immunofluorescence method to detect the localization of Rap-GTP. Description of data: supporting Figure S1 with legend: Capacitated, SLO-permeabilized sperm were incubated with 2-APB and EDTA as described under Methods. Cells were loaded with 40 μM GDP-β-S or GTP-γ-S in a buffer with low MgCl2 concentrations during 10 min at 37°C and bound nucleotides stabilized with 15 mM MgCl2 (5 min at 37°C). Cell suspensions were fixed in 2% paraformaldehyde, attached to poly-L-lysine coated coverslips, and overlain with 140 nM GST-Ral-GDS-RBD in blocking solution. Cells were triple stained with an anti-GST antibody as read out for the activity probe that detects active Rap (red, left panel), FITC-PSA (to confirm that the AR was effectively prevented by 2-APB; green, central panels), and Hoechst 33342 (to visualize all cells in the field; blue, right panels). Bars = 5 μm. [file 12964_2014_43_MOESM1_ESM.pdf]
