# Supplementary material for: Epac, Rap and Rab3 act in concert to mobilize calcium from sperm’s acrosome during exocytosis
Source: Cell Commun Signal. 2014 Aug 27;12:43. doi: 10.1186/s12964-014-0043-0 (PMC4156617; doi:10.1186/s12964-014-0043-0)
Supplement: Additional file 2: Figure S2. — Title of data: Primary antibodies raised in rabbits and introduced into sperm through SLO-generated pores do not interfere with the anti-GST read out in far-immunofluorescence experiments. SLO-permeabilized sperm were treated initially with 100 μM 2-APB, next with 134 nM anti-Epac or anti-Rap1 antibodies as indicated in the key, and finally with 0.5 mM CaCl2. Incubations were for 15 min at 37°C after each addition. The top two panels correspond to samples processed for Rap-GTP immunodetection as described under Methods (red, left panel). The remaining two panels correspond to samples overlain with Ral-GDS-RBD followed by anti-rabbit Cy3 but without anti-GST antibodies in between (red, left panel). Cells were stained with FITC-PSA (to confirm that the AR was effectively prevented by 2-APB; green, central panels) and Hoechst 33342 (to visualize all cells in the field; blue, right panels). Bars = 5 μm. [file 12964_2014_43_MOESM2_ESM.pdf]

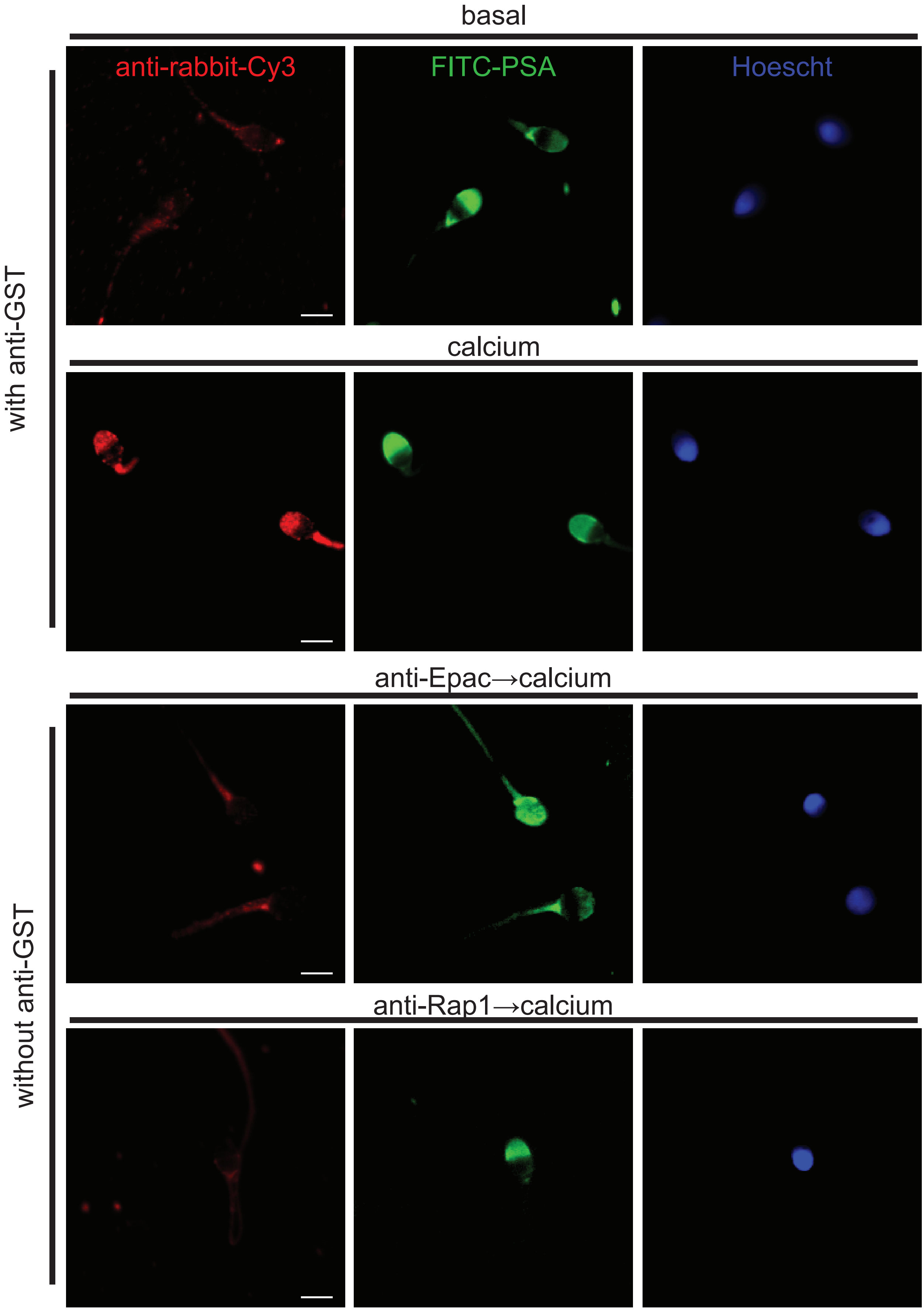
basal
anti-rabbit-Cy3
FITC-PSA
Hoescht
calcium
anti-Epac→calcium
anti-Rap1→calcium
with anti-GST
without anti-GST

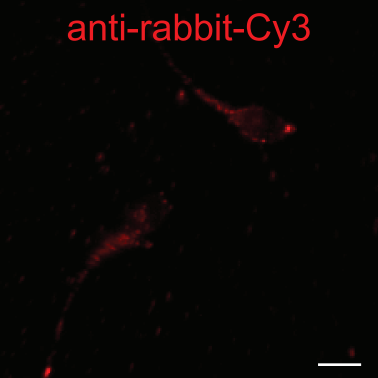
anti-rabbit-Cy3
